# Dietary patterns and changes in frailty status: the Rotterdam study

**DOI:** 10.1007/s00394-017-1509-9

**Published:** 2017-07-25

**Authors:** Sandra C. M. de Haas, Ester A. L. de Jonge, Trudy Voortman, Jolien Steenweg-de Graaff, Oscar H. Franco, M. Arfan Ikram, Fernando Rivadeneira, Jessica C. Kiefte-de Jong, Josje D. Schoufour

**Affiliations:** 1 Department of Epidemiology, University Medical Centre, Erasmus MC, P.O. Box 2040, 3000 CA Rotterdam, The Netherlands; 2 VU University Amsterdam, Amsterdam, The Netherlands; 3 Department of Internal Medicine, University Medical Centre, Erasmus MC, P.O. Box 2040, 3000 CA Rotterdam, The Netherlands; 4 Department of Global Public Health, Leiden University College The Hague, P.O. Box 13228, 2501 EE The Hague, The Netherlands

**Keywords:** Dietary patterns, Diet quality, Elderly, Frailty, Frailty index

## Abstract

**Purpose:**

To determine the associations between a priori and a posteriori derived dietary patterns and a general state of health, measured as the accumulation of deficits in a frailty index.

**Methods:**

Cross-sectional and longitudinal analysis embedded in the population-based Rotterdam Study (*n* = 2632) aged 45 years. Diet was assessed at baseline (year 2006) using food frequency questionnaires. Dietary patterns were defined a priori using an existing index reflecting adherence to national dietary guidelines and a posteriori using principal component analysis. A frailty index was composed of 38 health deficits and measured at baseline and follow-up (4 years later). Linear regression analyses were performed using adherence to each of the dietary patterns as exposure and the frailty index as outcome (all in *Z*-scores).

**Results:**

Adherence to the national dietary guidelines was associated with lower frailty at baseline (*β* −0.05, 95% CI −0.08, −0.02). Additionally, high adherence was associated with lower frailty scores over time (*β* −0.08, 95% CI −0.12, −0.04). The PCA revealed three dietary patterns that we named a “Traditional” pattern, high in legumes, eggs and savory snacks; a “Carnivore” pattern, high in meat and poultry; and a “Health Conscious” pattern, high in whole grain products, vegetables and fruit. In the cross-sectional analyses adherence to these patterns was not associated with frailty. However, adherence to the “Traditional” pattern was associated with less frailty over time (*β* −0.09, 95% CI −0.14, −0.05).

**Conclusion:**

No associations were found for adherence to a “healthy” pattern or “Carnivore” pattern. However, Even in a population that is relatively young and healthy, adherence to dietary guidelines or adherence to the Traditional pattern could help to prevent, delay or reverse frailty levels.

**Electronic supplementary material:**

The online version of this article (doi:10.1007/s00394-017-1509-9) contains supplementary material, which is available to authorized users.

## Introduction

Although there is no complete consensus on the conceptualization of frailty, experts agree that frailty is a state of increased vulnerability to adverse health outcomes [1]. The frailty index, developed by Mitnitski and Rockwood, appraises frailty as the accumulation of health-related and age-related deficits [2]. The included deficits cover a broad range of health aspects including cognition, disabilities, laboratory abnormalities, and comorbidities [3]. Several studies, among different age-categories and populations, show that a high frailty index score is associated with an increased risk for disability, falls, hospitalization, and mortality [4–7]. Prevention of frailty is important because it is difficult to recover from a frail state to a non-frail state [8]. One important modifiable factor that might either positively or negatively influences frailty is diet.

Most research on nutrition and frailty or overall health status has focussed on single nutritional components [9], such as macronutrients and micronutrients. Although these studies have provided valuable knowledge towards possible nutritional strategies to prevent frailty (e.g., high protein intake [10, 11], people do not eat single nutritional components but meals, combined into patterns. Dietary pattern approaches take into account the totality of the diet and allow for possible interactions and synergetic effects of nutritional components [12]. One way to define a person’s dietary pattern is via a priori approach, studying adherence to existing dietary guidelines or recommendations in relation to health outcomes. Alternatively, an a posteriori approach allows the identification of naturally occurring dietary patterns of populations [13]. The advantage of an a priori approach is that it allows for comparison between studies. The a posteriori approach has the advantage that can identify new dietary patterns, which could lead to improvements of current dietary guidelines. Taking into account both complementary approaches provides most insight into a possible association between dietary patterns and frailty.

Although a few previous studies evaluated dietary patterns and frailty, the majority of studies on frailty and nutrition use the frailty phenotype as an outcome [9, 14–17]. The frailty phenotype defines frailty as the presence of three out of five physical frailty symptoms (weight loss, weakness, exhaustion, slowness and low activity) [4]. Although this method has great advantages for clinical practice, due to its physical orientation, it is less useful as a measure of overall health [18]. A different, more holistic approach to frailty is the frailty index [2]. Information on how dietary patterns are associated with the frailty index is scarce. To our knowledge, only one previous study, by Woo et al., examined dietary patterns and the frailty index and found that better diet quality was associated with a lower frailty index [14]. Nevertheless, no longitudinal studies assessing the association between diet quality and changes in frailty index over time have been performed.

To provide more insight into how diet quality is associated with the frailty index and changes in frailty status over time we aim to: (1) examine the cross-sectional association between adherence to national dietary guidelines (a priori defined dietary pattern) and population-specific (a posteriori derived) dietary patterns and the frailty index in middle-aged and elderly populations and (2) examine if these a priori and a posteriori defined dietary patterns are associated with changes in frailty over a 4-year follow-up period.

## Methods

### Study population and design

This study was embedded in the Rotterdam Study (RS)–an ongoing prospective cohort in the Netherlands [19]. A more detailed description of the RS is provided elsewhere [19]. Briefly, the first baseline visits took place between 1990 and 1993. All residents aged 55 years and over in the Ommoord district of Rotterdam (*n* = 10,215), the Netherlands, were invited to participate, of which 7983 (78%) took part in the RS’s first cohort (RS-I). The study was extended in the year 2000 (RS-II; *n* = 3011) and in 2006, inviting all residents aged 45 years and over (RS-III; *n* = 3932). In total, 14,926 participants were included in the RS, who visited the research center for detailed measurements every 3–4 years. During an extensive home interview, trained research assistants collected data on a broad range of health variables including, activities of daily living, current health status, use of medication, depression and lifestyle. Subsequently, participants visited the study center for detailed examinations with an emphasis on imaging, collection of body fluids, and physical functioning. The RS was approved by the Medical Ethics Committee of the Erasmus Medical Center and by the review board of The Netherlands Ministry of Health, Welfare and Sports. All participants signed an informed consent. This study adheres to the Declaration of Helsinki for research involving human subjects.

For the current study, we included the first and second visit of the third cohort of the RS (RS-III-1and RS-III-2) comprising of 3932 participants. For 2632 participants, valid dietary intake and a frailty index were available at baseline (2006–2008) and for 2253 participants a frailty index was also available at follow-up (2012–2013, Fig. 1).

**Fig. 1:**
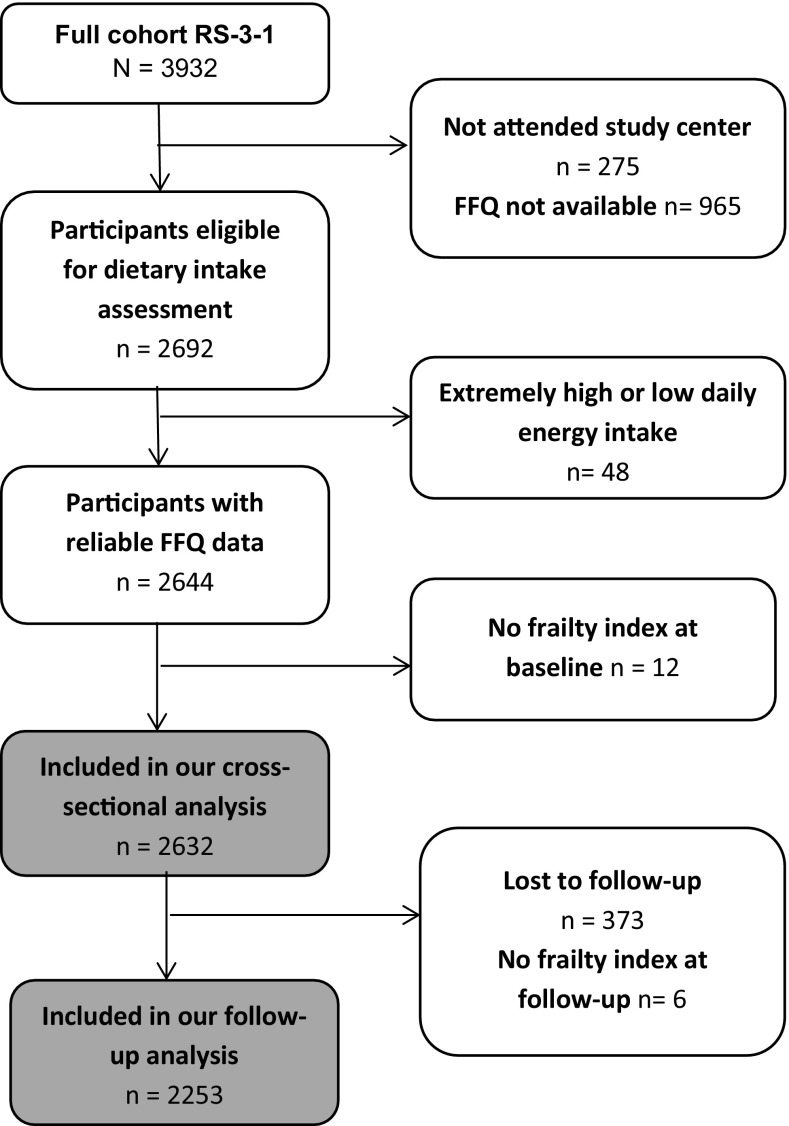
Flowchart of the study population. *FFQ* Food Frequency Questionnaire

### Dietary assessment

Dietary intake was measured with a self-administrated semi-quantitative food frequency questionnaire (FFQ) developed by Wageningen University and Research centre, adapted for the Rotterdam Study. The ability of the FFQ to rank people according to their intake was previously shown in two validation studies using a 9-day dietary record [20] and a 4-week dietary history [21]. The FFQ includes 389 items about the frequency and amount of consumed food items in days, weeks and months according to the previous year and was filled out at home. For the estimation of the portion sizes in grams standardized household measures were applied [22]. For calculation of the nutritional data the Dutch Food Composition Table (NEVO) of 2006 was used [23]. Participants with extremely high (>5000 kcal) or low (<500 kcal) daily energy intake were excluded as it was assumed that their questionnaire was unreliable (Fig. 1).

### Dietary patterns

Two different approaches to determine dietary patterns were applied: (1) an a priori defined index for diet quality and (2) a posteriori defined dietary patterns using principal component analysis (PCA).

#### A priori defined patterns and assignment of pattern adherence scores

We applied The Dutch Healthy Diet index (DHD-index), developed by van Lee et al. [24]. The DHD-index is a validated index, examining adherence to the Dutch Guidelines for a Healthy Diet of 2006 from the Dutch Health Council [25, 26]. The original DHD-index included ten guidelines based on the recommendations of the Dutch Health Council (Supplementary Table I). Participants received a sub-score, using a 10-point scale that reflected their adherence to each of these ten guidelines. These sub-scores were then summed to obtain a single index for each participant. No information was available on the use of fish oil capsules, so only dietary intake of fish was included. Due to limited information on acidic drinks and foods in our cohort, and because we were solely interested in the effect of diet, we created an adapted version of this original index excluding acidic drinks and physical activity, with a theoretical range of 0 till 80 points. A higher score represented higher adherence to the national guidelines.

#### A posteriori defined patterns and assignment of pattern adherence scores

All food items were categorized into 28 pre-defined food groups to reduce the complexity of dietary data. An overview of these food groups, which were based on similarities in product composition (for example lean versus fat dairy products) or culinary use (for example readymade meals), is shown in Supplementary Table II. Next, dietary patterns were derived by PCA on intake of these food groups in grams per day, unadjusted for total energy intake. We used varimax rotation and Kaiser Normalization to obtain patterns with simpler structure [27] and optimal interpretability. Factor loadings, which reflect the correlation between a food group and a dietary pattern, were used to characterize and label a pattern using a cut-off of 0.2. Food groups with a factor loading >0.2 indicate a positive contribution and <−0.2 a negative contribution to a specific pattern [46, 47]. Adherence to patterns with an Eigenvalue (a measure of explained variance) of >1.5 only was studied in relation to the frailty index. For each participant, pattern adherence scores (*Z*-scores) were constructed by summing up observed intakes of the pattern’s food groups weighted by the corresponding factor loading for each of the three dietary patterns separately.

### Frailty index

Frailty was measured with a frailty index, an instrument based on the accumulation of health deficits [2]. In general, deficits can be symptoms, signs, diseases, disabilities and laboratory measurements as long as they are age-related and health-related and are not too exceptional or too common [3]. We used a slightly adapted version of a previous validated frailty index designed for the Rotterdam Study, consisting of 38 health-related variables covering several health domains: functional status (*n* = 13), health conditions (*n* = 6), cognition (*n* = 6), diseases (*n* = 6), nutritional status (*n* = 3) and mood (*n* = 4) [28]. Deficits were dichotomized or categorized into a score ranging from 0 (not present) till 1 (present). Per person, the number of present deficits was divided by the total number of deficits, providing a continuous score ranging from 0 (no deficits present, least frail) till 1 (all deficits present, extremely frail). Missing values on the deficits were imputed using multiple imputation by chained equations [28]. Individuals with less than 20 observed items were determined to have insufficient information to considerably contribute to a valid frailty index and were excluded from the analyses (Fig. 1). To be able to evaluate changes over time we had to remove seven items from the original Rotterdam Study Frailty Index, namely: vitamin D, sex hormone binding globulin, mobility, uric acid, proBNP, CRP and homocysteine. Because, unfortunately, these biomarkers were not assessed at follow-up. Characteristics of the original Rotterdam Study frailty index and the adapted version are provided in Supplementary Table III, no major differences in the mean or median were observed. Furthermore, the two scales had a high mutual correlation (*r* = 0.98) and similar associations with age and mortality (Supplementary Table III).

### Covariates

Height (cm) and body weight (kg) were measured at the research center using a stadiometer wearing light clothing. Body mass index (BMI) was calculated as body weight (kg) divided by height (m)^2^. Smoking status was classified as never, former or current smoker. Level of education was determined by the highest attained education and classified as low (primary education and lower vocational education), middle (secondary general education and secondary vocational education), middle-high (higher general education) or high (higher vocational education or university education). Monthly household income was classified as low (<€1.500), middle (€1.500–2.900) or high (>€2.900). Physical activity was assessed with the LASA physical activity Questionnaire (LAPAQ) and metabolic equivalents (MET) scores were calculated as the sum of hours a week spent in light, moderate or vigorous activity (walking, cycling, gardening, sports, and hobbies), expressed in metabolic equivalent of task (MET) score [29]. MET scores represent the energy that is required for an activity divided by the energy necessary at rest [30]. Total energy intake in kilocalories per day and use of dietary supplements (yes/no) were all retrieved from the FFQ.

### Statistical analysis

First, baseline characteristics of the study population were shown in strata of frailty, dichotomized at the median. A *p* value for the observed values was provided using independent sample *t* tests for continuous variables and *X* ^2^ for categorical variables. Second, linear regression analyses were performed to examine the cross-sectional associations between adherence to each dietary pattern and the frailty index at baseline (all in *Z*-scores). Analyses were performed as a basic model, adjusted for age and sex (model 1), followed by a model that was additionally adjusted for smoking, level of education and income, physical activity, dietary supplement use (model 2), and a model additionally adjusted for energy intake (model 3). Confounders were tested based on previous studies [31, 31] and included in the models if they substantial change in effect-estimate on at least one of the dietary patterns (>10%). Additionally, the three a posteriori derived dietary patterns were adjusted for each other. The third models were adjusted for total energy intake because by design of the DHD-index, it might be easier to adhere to the guidelines at higher levels of energy intake. Third, we evaluated the association between the dietary patterns and changes in frailty index over time. To test if the frailty index changed significantly over time we applied a paired *t* test. Thereafter, in line with the cross-sectional results we created a basic model and an adjusted model, using the frailty index at follow-up as an outcome, additionally adjusting for the baseline frailty index. The coefficients of this model can be interpreted as the difference between the mean change frailty index score for each unit increase in exposure [32]. For the a posteriori defined dietary patterns we calculated the food group intakes corresponding to 1SD difference in dietary pattern adherence to increase the interpretability of the results. To exclude the possibility that results were driven by nutrition-associated deficits in the frailty index, we created a frailty index without BMI, HDL and total cholesterol and reran the analyses.

Additionally, we performed several sensitivity analyses using the cross-sectional data. To test potential selection bias, we calculated and compared the frailty index score for participants included and excluded in the main analyses. We tested for potential interaction by adding the product term of adherence to each of the dietary patterns with total energy intake to model 3. A similar approach was used to study interaction with sex, age and BMI. Stratified analyses were performed if the *p* for interaction was <0.05. Last, we performed the analyses in subgroups after excluding (1) participants with incomplete dietary intake data (>1% missing items in the FFQ), and (2) participants who deceased within 3 years after baseline. Analyses were performed using SPSS statistical software (IBM, version 23). A *p* value <0.05 was considered statistically significant.

## Results

### Subject characteristics

The median (interquartile range) frailty index of our population (*n* = 2632) was 0.14 (0.09, 0.19). Characteristics of our study population in strata of the frailty index above and below the median are shown in Table 1. On average, participants were 57 years (SD = 7.2) and the mean DHD-index for the full population was 56.2 (SD 9.28).

**Table 1:** Baseline characteristics of the study sample

	Low frailty index (≤the median^a^)	High frailty index (>the median)	*p* value^c^
*n*	1350	1282	
Baseline characteristics			
Sex (% men)	565 (42%)	534 (42%)	0.48
Age (years)^b^	55.9 (5.2)	58.1 (7.1)	<0.001
Smoking (%)			0.07
Never	447 (33%)	375 (29%)	
Former	575 (42%)	595 (46%)	
Current	328 (24%)	312 (24%)	
Income (%)			<0.001
Low	169 (13%)	296 (23%)	
Middle	543 (40%)	604 (47%)	
High	638 (37%)	382 (30%)	
Level of education (%)			<0.001
Low	257 (19%)	411 (32%)	
Middle	565 (42%)	506 (40%)	
High	525 (39%)	357 (28%)	
Alcohol use (glasses per day)	1.36 (1.47)	1.26 (1.68)	0.09
Supplement use (% yes)	646 (48%)	677 (53%)	0.01
Frailty index score	0.09 (0.03)	0.21 (0.03)	<0.001
Dutch Heathy Diet Index	56.6 (9.10)	55.9 (9.46)	0.067
Adherence to “Traditional” pattern (*Z*-scores)	0.06 (0.96)	−0.06 (0.95)	0.001
Adherence to “Carnivore” pattern (*Z*-scores)	−0.04 (0.89)	0.03 (0.96)	0.066
Adherence to “Health Conscious” pattern (Z-scores)	−0.03 (1.00)	0.05 (0.96)	0.025
BMI (kg/m^2^)	26.2 (3.65)	28.9 (4.98)	<0.001
Energy intake (kcal)	2334 (696)	2250 (737)	0.003
Physical activity: METh/week	61.6 (55.1)	53.9 (62.2)	<0.001

*BMI* body mass index, *METh* metabolic equivalent of task in hours

^a^ Our population- specific median is 0.14

^b^ Mean + SD

^c^ *p* value calculated using independent sample t-tests for continuous variables and *X* ^2^ for categorical variables

### Dietary patterns derived by principal component analysis

A posteriori, we derived three population-specific dietary patterns that we labeled: (1) a “Traditional” pattern, characterized by a high intake of savory snacks, legumes, eggs, fried potatoes, alcohol, processed meat and soup; (2) a “Carnivore” pattern, characterized by a high intake of red meat and poultry with a low intake of meat replacements; and (3) a “Health Conscious” pattern, characterized by a high intake of whole grains, vegetables, fruit and nuts. The factor loadings of the food groups are presented in Table 2. The “Traditional” pattern explained 10.0%, the “Carnivore” pattern 7.7% and the “Health Conscious” pattern 5.4% of the total variance in food group intake (Table 2). The DHD-index was positively associated with the “Traditional” pattern (Pearson’s *r* = 0.37) and with the “Health Conscious” pattern (Pearson’s *r* = 0.13), and negatively associated with the “Carnivore” pattern (Pearson’s *r* = −0.25).

**Table 2:** A posteriori defined dietary derived from principal component analysis

Food groups	“Traditional” pattern	“Carnivore” pattern	“Health Conscious” pattern
Whole grain products	^a^	^a^	0.76
Refined grain products	0.24	^a^	−0.44
Lean dairy products	^a^	^a^	0.27
Fat dairy products	^a^	^a^	^a^
Fruit	−0.25	^a^	0.42
Vegetables	^a^	^a^	0.50
Legumes	0.51	^a^	^a^
Potatoes	0.21	0.25	0.24
Fried potatoes	0.45	^a^	^a^
Poultry	^a^	0.48	^a^
Unprocessed red meat	^a^	0.65	^a^
Processed meat	0.33	0.60	^a^
Meat alternatives	0.24	−0.63	0.21
Eggs	0.47	^a^	^a^
Lean fish	^a^	^a^	^a^
Fatty fish	^a^	^a^	^a^
Readymade meals	^a^	^a^	^a^
Tea	^a^	^a^	0.28
Coffee	^a^	^a^	^a^
Water and diet soda	^a^	^a^	^a^
Sugar sweetened beverages	^a^	^a^	^a^
Alcohol	0.41	^a^	^a^
Sweet snacks	^a^	^a^	^a^
Savory snacks	0.59	0.23	^a^
Nuts	0.26	−0.21	0.39
Vegetable oils and spreads	0.20	^a^	^a^
Animal fats	^a^	^a^	^a^
Soup, sauce, gravy and dressing	0.32	0.22	^a^
Eigenvalue	2.8	2.2	1.5
Explained variance (%)	10.0	7.7	5.4

^a^ Food groups with a factor loading between −0.20 and 0.20 were not shown

### Cross-sectional results: associations between dietary pattern adherence and the frailty index

In the fully adjusted models, a priori defined adherence to the DHD-index was associated with lower frailty index scores (*β* (95% CI) = −0.07 (−0.10, −0.03), Table 3, model 3. This implies that with every SD increase in DHD-index (1 SD = 9.28) the frailty index was on average 0.05 SD lower (1 SD = 0.08). More specifically, any of the following was associated with a 0.08 lower frailty index score: 200 g of vegetables, 14 g fibers per 100 kcal a day or <1 energy % trans fatty acids (supplementary data Table 1). After adjustment for all covariates and energy intake, of the a posteriori pattern only the “Carnivore” pattern was associated with frailty (Table 3). The interpretation of the *Z*-scores (one SD difference) for each dietary pattern is provided in Supplementary Table V.

**Table 3:** Cross-sectional associations between adherence to dietary patterns and the frailty index at baseline (*n* = 2632)

Dietary pattern	Model 1		Model 2		Model 3		
	*β*	(95% CI)	*β*	(95% CI)	*β*		(95% CI)
A priori defined	Reflection of adherence to national dietary guidelines						
Dutch healthy diet index (DHDI)	**−0.08**	**(−0.12, −0.05)**	**−0.07**	**(−0.10, −0.03)**	**−0.07**		**(−0.10, −0.03)**
A posteriori defined	Reflection of population-specific dietary patterns						
Traditional pattern	**−0.04**	**(−0.08, −0.05)**	−0.00	(−0.04, 0.03)	0.01		(−0.03, 0.05)
Carnivore pattern	**0.05**	**(0.01, 0.09)**	0.04	(−0.00, 0.08)	**0.05**		**(0.01, 0.07)**
Health conscious pattern	0.02	(−0.01, 0.06)	0.03	(−0.01, 0.06)	0.03		(−0.01, 0.07)

Model 1: adjusted for age and sex

Model 2: adjusted for age, sex, smoking, level of education, income, physical activity, and supplement use

Model 3: adjusted for age, sex, smoking, level of education, income, physical activity, supplement use and total energy intake

Adherences to a posteriori defined patterns were additionally adjusted for each other

Regression coefficients represent the differences in frailty index at baseline (in *Z*-scores, one *Z*-score represent a frailty index score of 0.08) per *Z*-score increase in dietary pattern adherence

Bold values indicate the significance based on a *p* value of <0.05

### Longitudinal results: associations between dietary pattern adherence and changes in the frailty index

In total, 2253 participants were included in the longitudinal analyses. For these participants, the median frailty index at follow-up was 0.14 (SD = 0.08) and frailty index was 0.007 lower at follow-up than at baseline (SD = 0.06, *p* value <0.001). Higher adherence to the a priori defined DHD-index and the a posteriori “Traditional” pattern at baseline were associated with reduced frailty indices overtime in all models, *β* (95% CI) = −0.07 (−0.10, −0.04) and *β* (95% CI) = −0.07 (−0.11, −0.04), respectively, Table 4, model 3. These results imply that with any of the following the frailty index at follow-up was 0.005 lower: 21 g/day increase in refined grain products, 13 g/day increase in potatoes or 18 g/day increase in savory snacks (supplemental table V). Adherence to the a posteriori derived “Carnivore” pattern was associated with an increased frailty index overtime in model 2, *β* (95% CI) = 0.03 (0.00, 0.07), but these results were no longer significant if adjusted for energy intake. The a posteriori defined “Health conscious” pattern was not associated with changes in frailty.

**Table 4:** Longitudinal associations between adherence to dietary patterns and changes in the frailty index between follow-up and baseline (*n* = 2253)

Dietary pattern	Model 1			Model 2		Model 3	
	*β*	(95% CI)		*β*	(95% CI)	*β*	(95% CI)
A priori defined	Reflection of adherence to national dietary guidelines						
Dutch Healthy Diet Index (DHDI)	**−0.07**	**(−0.10, −0.04)**		**−0.07**	**(−0.10, −0.03)**	**−0.07**	**(−0.10, −0.04)**
A posteriori defined	Reflection of population-specific dietary patterns						
Traditional pattern	**−0.08**	**(−0.11, −0.05)**		**−0.07**	**(−0.11, −0.04)**	**−0.07**	**(–0.11, −0.04)**
Carnivore pattern	**0.04**	**(0.01, 0.08)**		**0.03**	**(0.00, 0.07)**	0.04	(−0.01, 0.07)
Health conscious pattern	0.01	(−0.03, 0.03)		0.01	(−0.03, 0.04)	0.01	(−0.03, 0.04)

Model 1: adjusted for age, sex and baseline frailty index (in *z*-scores)

Model 2: adjusted for age, sex, baseline frailty index (in *z*-scores), smoking, level of education, income, physical activity, and supplement use

Model 3: adjusted for age, sex, baseline frailty index (in *z*-scores), smoking, level of education, income, physical activity, supplement use, and total energy intake

Adherences to a posteriori defined patterns were additionally adjusted for each other

Regression coefficients represent the differences in frailty index over the follow-up period (in *Z*-scores, one *Z*-score represent a frailty index score of 0.06) per *Z*-score increase in dietary pattern adherence

Bold values indicate the significance based on a *p* value of <0.05

### Sensitivity analyses

We observed that excluded participants had on average a higher frailty index score (mean 0.18) than the included participants (mean 0.14, *p* value <0.001). Additional adjustment by BMI did not highly influence the results. However, the cross-sectional analyses between adherence to the “Carnivore” pattern with frailty was no longer significant. We did not observe significant interaction terms between any of the dietary patterns and gender (*p* value range 0.13–0.86), total energy intake (*p* value range 0.06–0.60) or BMI (*p* value range 0.27–0.94) on frailty. Supplementary Table IV shows the sensitivity analyses for the cross-sectional results. Using the original 45-item frailty index did not influence the association between the a priori derived DHD-index and frailty (Supplementary Table IV). Excluding participants that deceased within 3 years (*n* = 38) or participants with incomplete FFQ data (*n* = 867) provided similar results for the a priori derived DHD-index, whereas the a posteriori derived “Carnivore” pattern was significantly associated with higher frailty scores when excluding participants who died within 3 years and participants without fully complete FFQs (Supplementary Table IV). Last, although effect estimates were similar, we observed a slightly stronger association between the DHDI index and frailty in participants aged above the median (57 years) than in those below the median age (Supplementary Table IV).

## Discussion

In this population-based cohort of middle aged and elderly persons, we observed that higher adherence to an a priori defined healthy dietary pattern was associated with lower frailty index at baseline, and with beneficial changes in frailty during follow-up. Furthermore, adherence to the a posteriori defined “Traditional” pattern was associated with lower frailty index over time.

Data on the association between nutrition and frailty are scarce and direct comparison of our results with published data is challenging for several reasons. First, other studies used different definitions of frailty or overall health. For example, social health, self-perceived health and resilience are identified to be important for healthy aging and are, therefore, included in several healthy aging instruments, but are not part of the frailty index [33]. Additionally, studies regarding frailty and dietary patterns use the frailty phenotype, defined as the presence of three out of five physical frailty symptoms: weight loss, self-reported exhaustion, weakness, slow walking speed, and low physical activity [4]. The frailty phenotype is physically orientated and is distinct from disabilities, chronic diseases, cognition and mental health, whereas the frailty index does includes these health domains. Second, national dietary guidelines and population-specific dietary patterns differ per country and per study population, as they are shaped by local or cultural habits and availability of food products [13].

Overall, previous studies found inconsistent results regarding the association between a priori defined dietary patterns and frailty, or aspects of frailty. To our knowledge, Woo et al. [14] are the only ones to report on the association between the frailty index and an a priori defined dietary pattern. They found that adherence to the Diet Quality Index International (DQI-I), an index based on (1) overall food group variety, (2) adequacy of vegetables, fruit, grains, fiber, protein, iron, calcium and vitamin C, (3) moderation of total fat, saturated fat, cholesterol, sodium and empty calorie foods, and (4) overall balance in macronutrient intake and fatty acid ratio [34] was associated with lower frailty index. However, they did not adjust for other lifestyle factors or total energy intake. In line with our results, several, but not all, studies indicate that adherence to national or international dietary guidelines might beneficially affect (physical) frailty [9]. Samieri et al., found a positive association between diet quality and overall health [35], whereas Akbaraly et al., did not identify a positive association between diet quality and overall health [36]. Furthermore, several papers report that adherence to a healthy diet (defined by different dietary guidelines) is generally associated with better cognitive functioning, less depressive symptoms and better physical functioning [9], all components of the frailty index. In addition to adherence to dietary guidelines, adherence to the Mediterranean diet has been observed to have several beneficial effects on health outcomes [37]. Previous efforts observed that the Mediterranean diet score was inversely associated with the prevalence of physical frailty [15], and a lower incidence of physical frailty [16, 17].

Recently, Assmann et al. studied overall health and its association with a posteriori defined dietary patterns in a French elderly population. They defined healthy aging as: not developing any major chronic diseases, good physical, and cognitive function, no limitations in IADL, no depressive symptoms, no health-related limitations in social life, good overall self-perceived health, and no function-limiting pain in a 13-year follow-up period. They found that a healthy dietary pattern (characterized by high intake of micronutrients, fibers and antioxidants) was associated with better health, but only among subjects with low energy intake [38]. In addition, “Health Conscious” or “Prudent” patterns did show associations with different aspects of healthy aging including self-perceived health, cognition and depression [9, 40–43]. Contrary to our expectations, we did not find an association between the “Health Conscious” pattern and the frailty index. This non-significant association could be explained by the relatively low explained variance of our “Health Conscious” pattern (5.4%). Previously, an a posteriori defined dietary pattern high in meat and fatty foods showed an inverse association with overall health, defined as maintaining a good mental health with the absence of major chronic diseases and limitations in physical functioning [43]. This pattern shows similarities with the “Carnivore pattern” in our population. Nevertheless, we did not find an association between this pattern and frailty when models were adjusted for socio-economic factors and lifestyle.

Our sensitivity analysis showed that adherence to the dietary guidelines (DHD-index) was associated with less frailty independent of BMI, HDL and total cholesterol, which are established intermediate health factors associated with dietary intake. We could speculate that an overall healthy diet can influence frailty via several mechanisms and pathways (e.g., diseases, cognition), not only via deficits directly associated with nutrition. The effect estimates observed in our study were rather small. For example, the (unadjusted) association between adherence to the “Carnivore” pattern and frailty implies that the frailty index is 0.004 points higher with every SD increase in dietary pattern adherence, which can be any of the following: 8 grams more poultry, 16 g more unprocessed red meat or 19 g more processed meat (Supplementary Table IV). Nevertheless our results show that adherence to a healthy diet can contribute to a better overall health status and better potential to preserve this health status during a 4-year follow-up period, which can have important implications on a population level.

Our study has several strengths. First, our combined use of a priori and a posteriori defined dietary patterns provided an opportunity to study both adherence to existing guidelines and population-specific patterns, in relation to frailty. Whereas the first approach provided us insight into the potential of current dietary guidelines to prevent frailty, the latter could provide additional insight to improve these guidelines in the future. Both methods have their own strengths. Whereas the PCA-derived dietary patterns are data driven and consider the correlation structure between food groups, the pre-defined index could be used to quantify a participant’s dietary quality, regardless of its source population. The latter facilitates comparisons between populations [48]. Furthermore, we were able to establish longitudinal associations between dietary pattern adherence and changes in frailty. This is a strength, because in a cross-sectional design it is not possible to state if participants became more frail as a consequence of their dietary pattern or if they adapted their dietary pattern due to their frailty status [44]. Additionally, we excluded participants that deceased within the first 3 years of follow-up in a sensitivity analyses. Together, this implies that the found results are true associations and not a result of reversed causation.

Nevertheless, we also recognize some limitations. Participants had relatively low frailty indices (e.g., low variation), which could result in less pronounced associations. We observed that on average the frailty index became lower over time, whereas it was expected to increase. Similarly, weaker or frailer elderly people may be less able or willing to come to the study center and/or fill in the extensive FFQ [44, 45], which might have led to selection bias. Indeed, we observed a higher frailty index score for excluded participants than for included participants. Furthermore, definition and labeling of the a posteriori defined patterns involved some arbitrary choices, including the definition of food groups, and the cut-off values of factor loadings and Eigenvalues. Additionally, the dietary patterns identified only explained 20% of the variance of the total diet, reflecting the complexity of reducing the variation in dietary intake data into single components. Last, the interpretation of a posteriori defined dietary patterns can be difficult. To increase comparability between the a priori defined dietary pattern and the a posteriori defined dietary patterns we provided all estimates in *z*-scores. To improve the interpretation of the estimates we calculated the food group intakes corresponding to 1SD difference in dietary pattern adherence (supplemental Table V).

In conclusion, in this population of middle-aged and elderly participants, we observed that population-specific dietary patterns were not consistently associated with frailty or changes in frailty status over time. Adherence to dietary guidelines was consistently associated with less frailty and a reduction of frailty over time. These results suggest that adherence to the Dutch dietary guidelines can help to prevent frailty in older adults and elderly people.

## Electronic supplementary material

Below is the link to the electronic supplementary material.

Supplementary material 1 (DOCX 49 kb)
