# Supplementary material for: Dietary patterns and changes in frailty status: the Rotterdam study
Source: Eur J Nutr. 2017 Jul 25;57(7):2365–75. doi: 10.1007/s00394-017-1509-9 (PMC6182690; doi:10.1007/s00394-017-1509-9)
Supplement: Supplementary file 1 — Supplementary material 1 (DOCX 49 kb) [file 394_2017_1509_MOESM1_ESM.docx]

**Supplementary data**

| Supplementary Table I: Scoring system of The Dutch Healthy Diet Index | | |
| --- | --- | --- |
| **Component** | **Minimum score**  **= 0 points** | **Maximum score**  **= 10 points** |
| 1. Physical activity | 0 activities a week | ≥ 5 activities a week |
| 2. Vegetables | 0 gram a day | ≥ 200 grams a day |
| 3. Fruit and fruit juices | 0 gram a dag | ≥ 200 grams a day |
| 4. Fibers | 0 gram per 1000 calories a day | ≥ 14 grams per 1000 kcal a day |
| 5. Fish and fish oil capsules | 0 milligram EPA and DHA a day | ≥ 450 milligrams EPA and DHA a day |
| 6. Saturated fatty acids | ≥ 16.6 energy% a day | < 10 energy% a day |
| 7. Trans fatty acids | ≥ 1.6 energy % a day | < 1 energy% a day |
| 8. Acidic drinks and foods | > 7 occasions a day | ≤ 7 occasions a day |
| 9. Sodium | ≥ 2.45 grams a day | < 1.68 grams a day |
| 10. Alcohol (ethanol) | Male: ≥ 60 grams a day  Female: ≥ 40 grams a day | Male: ≤ 20 grams a day  Female: ≤ 10 grams a day |

EPA = eicosapentaenoic acid. DHA = docosahexaenoic acid.

| Supplementary Table II: Food items included in each food group, used for the PCA analyses | |
| --- | --- |
| **Food group** | **Summary of included products** |
| 1. Whole grain products | Muesli, whole wheat bread, multigrain bread, brown rice |
| 1. Refined grain products | Cereals, white bread, ginger bread, white pasta |
| 1. Lean dairy products | Skim milk, low fat yoghurt, low-fat cheese, buttermilk |
| 1. Fat dairy products | Full milk, full-fat cheese, mousse, whipped cream |
| 1. Fruit | Apple, banana, orange, strawberry etc. |
| 1. Vegetables | Cauliflower, carrot, lettuce, tomato, etc. |
| 1. Legumes | Kidney beans, white beans, soup with legumes |
| 1. Potatoes | Boiled potatoes, mashed potatoes |
| 1. Fried potatoes | Frites, fried potatoes, baked potatoes |
| 1. Poultry | Chicken, turkey |
| 1. Unprocessed red meat | Cooked liver, meatloaf, beef, lamb |
| 1. Processed meat | Liver products, ham, hamburger, bacon |
| 1. Meat alternatives | Products based on tofu or tempeh, or other meat alternatives |
| 1. Eggs | Boiled eggs, baked eggs |
| 1. Lean fish | Mussels, cod, trout, crab |
| 1. Fatty fish | Herring, salmon, mackerel |
| 1. Readymade meals | Pizza, pancakes |
| 1. Tea | Black tea, green tea, herbal thee |
| 1. Coffee | Coffee |
| 1. Water and diet soda | Water, diet soda |
| 1. Sugar sweetened beverages | Orange juice, other fruit juices, soda, lemonade, water ice |
| 1. Alcohol | Beer, red wine, white wine, strong alcoholic beverages |
| 1. Sweet snacks | Sugar, cookies, chocolate, candy bars, candy |
| 1. Savory snacks | Fried fish, mini snack products, chips, peanuts, crackers |
| 1. Nuts | Pine nuts, walnuts, mixed nuts, linseed |
| 1. Vegetable oils and spreads | Low fat margarine, margarine, liquid margarine, olive oil |
| 1. Animal fats | Butter, solid cooking fat, solid frying fat |
| 1. Soup, sauce, gravy and dressing | Soups without legumes, mayonnaise, ketchup, tomato sauce |

Supplementary III

**Comparison between the original frailty index and the adapted frailty index**

The original Rotterdam Study Frailty Index was designed and validated among 11,539 participants of the Rotterdam Study. Construct validity (i.e. skewed distribution, correlation with age and sub-maximum score) and criterion validity were shown [1]. This frailty index consisted of 45 health-related variables, related to mood, cognition, functional status, diseases and conditions, biomarkers, and nutritional status. A total FI-score was calculated by averaging the scores of the deficits, resulting in a score between 0 and 1 with increasing frailty.

To be able to evaluate changes over time we had to remove seven items from the original Rotterdam Study Frailty Index, namely: vitamin D, sex hormone binding globulin, mobility, uric acid, proBNP, CRP, and homocysteine. Unfortunately, these biomarkers were not assessed at follow-up. It has been suggested to use the exact same variables if transitions or changes of frailty over time are evaluated [2]. Therefore we created an adapted Rotterdam Study frailty index, consisting of 38 health-related variables, related to mood, cognition, functional status, diseases and conditions, biomarkers, and nutritional status. Characteristics of the original Rotterdam Study Frailty Index and the adapted version are provided below, calculated among 11,539 participants of the Rotterdam study.

| **Characteristics** |  |  |
| --- | --- | --- |
| Mutual correlation (r) | 0.977 |  |
| Paired T-test: mean difference | 0.0018 |  |
| Paired T-test: t | 9.44 |  |
| Paired T-test: df | 11538 |  |
| Paired T-test: p-value | <0.001 |  |
|  | **Original Rotterdam Study FI** | **Adapted Rotterdam Study FI** |
| Mean (SD) | 0.180 (0.10) | 0.178 (0.10) |
| Medan (IQR) | 0.161 (0.11-0.22) | 0.160 (0.11-0.23) |
| Correlation age | 0.53 | 0.48 |
| Hazard Ratio (95%CI) | 1.051 (1.047-1.054) | 1.045 (1.041-1.048) |

Supplementary Table IV: Sensitivity analyses on cross-sectional results

|  |  | | **Excluding:** | | | | **Stratified by age category** | | | |
| --- | --- | --- | --- | --- | --- | --- | --- | --- | --- | --- |
|  | **Using the original 45-item frailty index** | | **Participants that died within 3 years (n=38)** | | **Participants with incomplete FFQ data (n=867)** | | **Participants aged ≤median age** | | **Participants aged >median age** | |
| **Dietary pattern** | **β** | **(95 % CI)** | **β** | **(95 % CI)** | **β** | **(95 % CI)** | **β** | **(95 % CI)** | **β** | **(95 % CI)** |
| Dutch Healthy Diet Index | **-0.06** | **(-0.09, -0.04)** | **-0.05** | **(-0.08, -0.01)** | **-0.09** | **(-0.13, -0.04)** | -0.04 | (-0.08, 0.01) | **-0.09** | **(-0.15, -0.04)** |
| Traditional pattern | -0.01 | (-0.04, 0.03) | -0.01 | (0.03, 0.04) | -0.04 | (0.08, 0.00) | 0.01 | (-0.04, 0.06) | 0.02 | (-0.04, 0.08) |
| Carnivore pattern | **0.04** | **(0.01, 0.08)** | **0.03** | **(0.01, 0.07)** | **0.05** | **(0.00, 0.10)** | 0.04 | (-0.01, 0.09) | 0.05 | (-0.01, 0.11) |
| Health conscious pattern | 0.02 | (-0.01, 0.05) | 0.02 | (-0.02, 0.05) | 0.02 | (-0.02, 0.06) | **0.05** | **(0.01, 0.09)** | -0.01 | (-0.06, 0.05) |

All models are adjusted for age, sex, smoking, level of education, income, physical activity, supplement use, and energy intake. Additionally the three a posteriori defined patterns are adjusted for each other. Regression coefficients represent the differences in frailty index (in Z-scores) per Z-score increase in dietary pattern adherence.

Median age for this population was 57 years.

| **Supplemental Table V: Food group intakes corresponding to 1 SD difference in dietary pattern adherence** | | | | | | | | | | | | |
| --- | --- | --- | --- | --- | --- | --- | --- | --- | --- | --- | --- | --- |
|  |  |  | | | **Traditional pattern** | | | **Carnivore pattern** | | | **Health conscious pattern** | |
| **Food groups** | | | **Mean (g/day)** | **SD**  **(g/day)** | **FL** | | **Difference per SD (g/day)^**^** | **FL** | | **Difference per SD (g/day)^**^** | **FL** | **Difference per SD (g/day)^a^** |
| Whole grain products | | | 94 | 78 | * | * | | * | * | | 0.76 | 59 |
| Refined grain products | | | 109 | 87 | 0.24 | 21 | | * | * | | -0.44 | -38 |
| Lean dairy products | | | 283 | 265 | * | * | | * | * | | 0.27 | 72 |
| Fat dairy products | | | 67 | 103 | * | * | | * | * | | * | * |
| Fruit | | | 347 | 317 | -0.25 | -79 | | * | * | | 0.42 | 133 |
| Vegetables | | | 280 | 192 | * | * | | * | * | | 0.50 | 96 |
| Legumes | | | 24 | 35 | 0.51 | 18 | | * | * | | * | * |
| Potatoes | | | 70 | 60 | 0.21 | 13 | | 0.25 | 15 | | 0.24 | 14 |
| Fried potatoes | | | 18 | 21 | 0.45 | 10 | | * | * | | * | * |
| Poultry | | | 19 | 18 | * | * | | 0.48 | 8 | | * | * |
| Unprocessed red meat | | | 35 | 25 | * | * | | 0.65 | 16 | | * | * |
| Processed meat | | | 43 | 32 | 0.33 | 11 | | 0.60 | 19 | | * | * |
| Meat alternatives | | | 4 | 15 | 0.24 | 4 | | -0.63 | -9 | | 0.21 | 3 |
| Eggs | | | 16 | 14 | 0.47 | 7 | | * | * | | * | * |
| Lean fish | | | 13 | 17 | * | * | | * | * | | * | * |
| Fatty fish | | | 10 | 16 | * | * | | * | * | | * | * |
| Readymade meals | | | 14 | 18 | * | * | | * | * | | * | * |
| Tea | | | 257 | 283 | * | * | | * | * | | 0.28 | 79 |
| Coffee | | | 413 | 269 | * | * | | * | * | | * | * |
| Water and diet soda | | | 229 | 283 | * | * | | * | * | | * | * |
| Sugar sweetened beverages | | | 103 | 125 | * | * | | * | * | | * | * |
| Alcohol | | | 163 | 238 | 0.41 | 98 | | * | * | | * | * |
| Sweet snacks | | | 67 | 48 | * | * | | * | * | | * | * |
| Savory snacks | | | 36 | 30 | 0.59 | 18 | | 0.23 | 7 | | * | * |
| Nuts | | | 6 | 12 | 0.26 | 3 | | -0.21 | -3 | | 0.39 | 5 |
| Vegetable oils and spreads | | | 21 | 23 | 0.20 | 5 | | * | * | | * | * |
| Animal fats | | | 4 | 10 | * | * | | * | * | | * | * |
| Soup, sauce, gravy and dressing | | | 63 | 64 | 0.32 | 20 | | 0.22 | 14 | | * | * |

Abbreviations: FL= factor loading; the standardized correlation coefficient between the dietary pattern and the food group. N.S: Non-significant associations between a dietary patter and bone outcome.

* Food groups with a factor loading between -0.20 and 0.20 were not shown.

^a^ The difference in intake per food group are calculated by multiplying the corresponding factor loading by the value of one SD of that food group in grams per day

[1] J.D. Schoufour, N.S. Erler, L. Jaspers, J.C. Kiefte-de Jong, T. Voortman, G. Ziere, J. Lindemans, C.C. Klaver, H. Tiemeier, B.H. Stricker, M.A. Ikram, J.S.E. Laven, G.G. Brusselle, F. Rivadeneira, O.H. Franco, Design of a frailty index among community living middle-aged and older people: The Rotterdam Study, Maturitas in press (2017).

[2] S.D. Searle, A. Mitnitski, E.A. Gahbauer, T.M. Gill, K. Rockwood, A standard procedure for creating a frailty index, BMC Geriatr 8 (2008) 24.
